# Mouse breeding facilities in Argentina: Current state, challenges, and strengths in relation to animal welfare

**DOI:** 10.3389/fvets.2022.1031976

**Published:** 2022-10-20

**Authors:** Agustina Resasco, Silvina Laura Diaz

**Affiliations:** ^1^Instituto de Biología Celular y Neurociencia (UBA - CONICET), Facultad de Medicina, Universidad de Buenos Aires, Ciudad Autónoma de Buenos Aires, Argentina; ^2^Laboratorio de Animales de Experimentación (LAE), Facultad de Ciencias Veterinarias, Universidad Nacional de La Plata, La Plata, Argentina; ^3^Cátedra de Técnica para Bioterio, Facultad de Farmacia y Bioquímica, Universidad de Buenos Aires, Ciudad Autónoma de Buenos Aires, Argentina

**Keywords:** mice, animal facility, animal welfare, Latin America, Argentina, breeding

## Abstract

The science and technology of laboratory animals has come a long way worldwide, but for reasons related to the development of the countries, this journey started later in some Latin American countries, as is the case of Argentina. Without a specific legal framework to conduct animal experimentation, local strengths to promote animal welfare are based on professionals specifically trained in the care of laboratory animals as well as an extended network of ethics committees that ensures compliance with the ethical principles applied to animal experimentation. Nevertheless, there are no updated reports showing welfare indicators in rodent facilities. Therefore, we conducted a survey on mice breeding facilities enrolled in a national record elaborated by the National Ministry of Science. Questions related to four of the Five Domains Model of Mellor, concerning (1) nutrition, (2) physical environment, (3) health, and (4) behavioral interactions with the environment, other animals, and humans, were included as well as information concerning general aspects of the establishments. Data obtained from 25 mice breeder facilities localized all over the country were summarized, providing for the first time a clear picture of the national situation about the welfare of laboratory mice in these establishments. This data will be essential to design future policy as well as for deciding priorities aiming to improve the welfare of mice bred in Argentinian facilities.

## Introduction

In most countries, particularly in developed countries from the global north, minimum requirements for laboratory animals are strictly regulated by specific legislation (1). For example, the UK passed the Animals (Scientific Procedures) Act in 1986, the member states of the Council of Europe must follow Directive 2010/63/EU on the protection of animals used for scientific purposes, the US follows the Guide for the Care and Use of Laboratory Animals (2), while in Canada legislation regarding animal research falls under provincial jurisdiction (3). Particularly, Latin America, Brazil, Mexico, and Uruguay have recently implemented specific legislation regarding the use of laboratory animals. Nevertheless, the current picture in this part of the globe is heterogeneous and frequently linked to the economic and political status of each particular country (4).

In Argentina, the situation of Laboratory Animal Science is not disconnected from the state of affairs in the country. In this sense, we have recently identified the strengths and difficulties in sight of the development of this scientific discipline (5). Regarding the legislation, Argentina has a law that protects animals against cruelty acts (Law 14346, proclaimed in 1954) but does not have a specific law that regulates scientific procedures in laboratory animals. Moreover, the modern view considers that to promote an optimal welfare state, minimum requirements must be surpassed by including appropriate refinements to the housing or husbandry protocols (1). Despite this difficult scenario, one can be optimistic because the standards for animals used in experiments conducted in Argentina are set by an extensive network of institutional ethics committees that oversee the experimental protocols in accordance with international recommendations (5). Nevertheless, the requirements for breeding laboratory animals are less regulated, which is problematic since in countries with specific regulations and statistics, at least three additional animals are needed for every two animals employed in experimentation (6).

One of the main drawbacks of not having a specific law that oversees animal experimentation is that there are no local statistics, so the extent to which modern refinements have been incorporated into the different animal facilities is currently unknown. Hence, the main objective of the present work is to characterize the current situation of the breeding facilities in Argentina, in order to identify its strengths as well as areas in which animal welfare might be compromised.

The first step is to define how to evaluate the welfare of the laboratory mice in the breeding facilities. In general, Animal Welfare Science aims to assess, through objective indicators, the subjective perception that an animal has of its own quality of life (7). This is clearly challenging since it involves the selection of appropriate markers across scientific disciplines (8), which can even include indirect markers such as those related to the environment. To address this problem, a framework based on five domains was first proposed by Mellor in 1994 (9), which was frequently revised and extended afterwards to ensure that the recommendations were up to date with the latest literature (10). Succinctly, the five domains model currently comprises: (1) Nutrition, including the quality and availability of the food and water supply; (2) Physical Environment, which consists of the enclosure's characteristics per se as well as the quality of the resources such as the air, light, and noise; (3) Health, considering disease due to pathological agents, poisoning, husbandry/experimental procedures that may cause pain or discomfort, among other things; (4) Positive and negative behavioral interactions with the environment, with other animals, and with humans. (5) Mental state, i.e., the affective processes derived from the previous four domains (e.g., feeling hungry due to an inappropriate supply of food). The first four domains can be assessed by direct observation of the animals or their environments, while the fifth domain would require specific assays to measure them indirectly [e.g. judgment bias task to assess positive or negative affective states triggered by enriched or standard housing, respectively (11)]. Therefore, we developed a questionnaire based on these first four domains to characterize, as described above, the breeding facilities in Argentina. Our samples were the institutions enrolled in the ‘Sistema Nacional de Bioterios' (SNB), a national record of animal facilities set by the National Ministry of Science, to which establishments adhere voluntarily. Overall, we expect that this information allows us to depict the actual animal welfare situation, as well as to propose future strategies to improve animal welfare, according to the reality of the region.

## Materials and methods

### Sample

We targeted institutions breeding mice that are voluntarily enrolled in the SNB, a national record of animal facilities set by the National Ministry of Science. Establishments adhere voluntarily to this registry, which allows them to access specific funding schemes. We identified 46 candidate institutions, and they were contacted *via* the email address that was noted in the national registry.

### Survey

The survey was conducted in Google Forms, consisting of 75 questions divided into five sections, and institutions participated on a voluntary basis. The full translated version can be seen in the Supplemental materials but briefly, the first section encompassed general questions about the size of the facility, species that they breed, type of records, genetic origin, and quality of the mouse colony. The remaining four sections were based on Mellor's Five Domains Model (10), considering the items that could be assessed by direct observation of the animals or their environment. First, the Nutrition domain was assessed, determining whether food and water were freely available, if these were treated to reduce the risk of microbiological contamination, if conditions such as over- or underweight are frequently observed [measured with the body condition scoring scale, a system that was adapted for the laboratory mouse (12)], and if unforeseen events, such as empty water bottles, have been recently detected. The following section considered the Environment, inquiring about the type of housing that is used, the control over the environmental conditions (such as the room temperature), the basic resources that are provided to the animals, the adverse effects of these resources, and the capacity of the institution to resolve unforeseen events (e.g. due to the presence of contingency plans). The third segment was about the health status of the colony. Here, we asked about microbiological monitoring, the presentation of certain health conditions, as well as preventive treatments. Finally, the behavioral interaction with the environment (in particular about the administration of environmental enrichment), with other animals, and with the personnel were assessed (the type of training/continuous education of the workers, the methods for handling, and the consequences of these interactions, i.e., if biting happened recently). The collected data was summarized and anonymously reported in the Results, according to the different five sections.

## Results

Ten out of the 46 institutions enrolled in the SNB did not answer the survey. Of the remaining 36 institutions, 11 were not included in the results since they declared that they do not breed laboratory mice. Therefore, for the data analysis, 25 complete forms from institutions that breed mice were processed. Results are presented according to the five sections of the survey.

### General description

**Figure 1:**
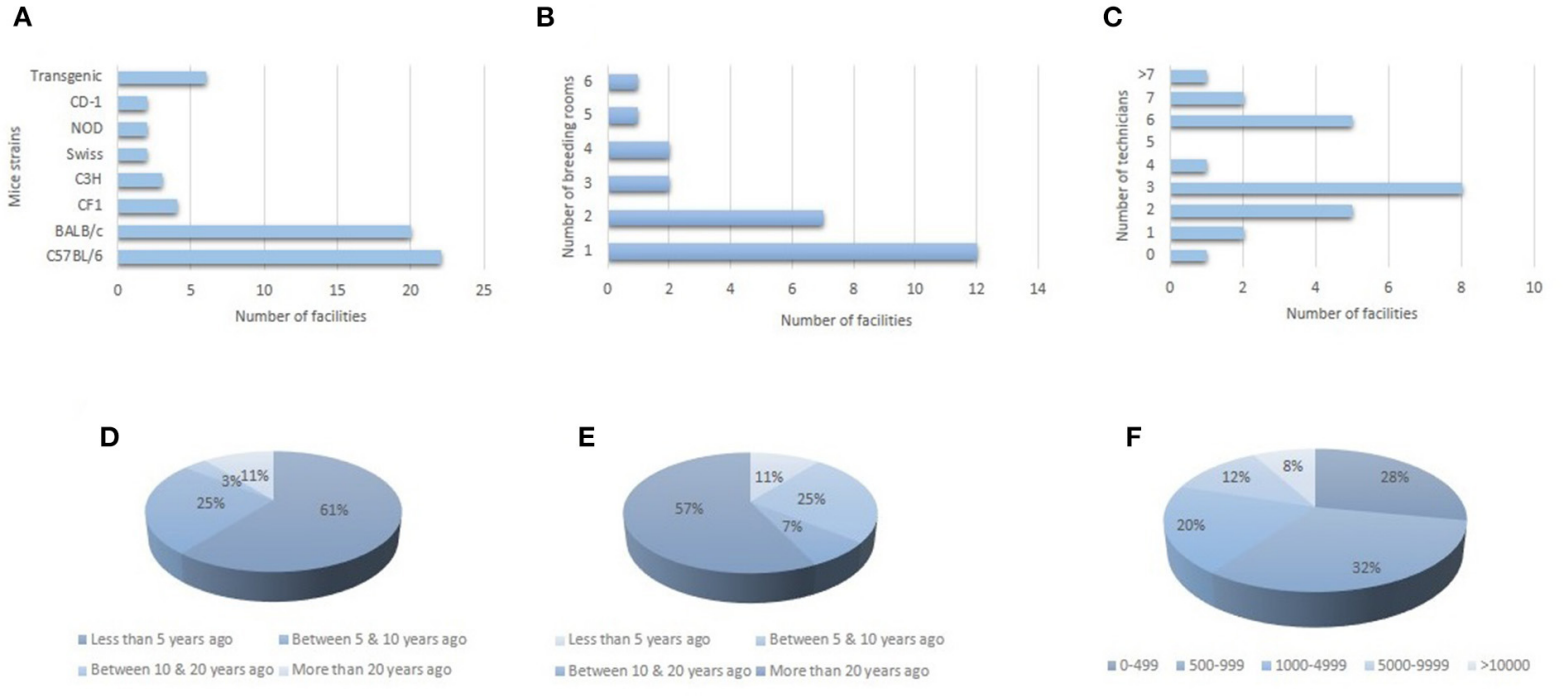
Characteristics of the breeding colonies and facilities, including the number of people devoted to the care of the animals. **(A)** Frequency of inbred strains and outbred stocks in the different institutions. **(B)** Number of rooms that facilities devote to breeding mice. **(C)** Number of technicians available to supervise mice. **(D)** Date of first acquisition of breeders. **(E)** Real age of mice strains, taking into account when breeders were first imported to the country. **(F)** Number of mice produced per year in each institution.

The laboratory mouse is the sole species bred in 11 institutions, whereas half of the facilities that filled the form breed rats besides mice. Additionally, six facilities breed less common species (e.g. rabbits). The most popular strains among the 25 institutions (Figure 1A) are related to the C57BL/6 and the BALB/c families. Indeed, C57 and BALB/c mice are bred in 88% and 80% of the facilities, respectively. However, in most of them, proper nomenclature or even the origin of mice, are not properly defined/known. Concerning available outbred stocks, CF1, Swiss, and NOD are present in four, two, and two facilities, respectively. Transgenic lines are also bred in six institutions. Most institutions have one or two rooms specifically devoted to mice breeding (Figure 1B), whereas the number of technicians devoted to the work with animals is quite variable among the institutions (Figure 1C). In 64% of the facilities, technicians are specifically devoted to animal-related labor, whereas in the rest, technicians rotate between different tasks. Except for one facility, single species are maintained in the same room, but in most of them (80%), different mice strains are kept in the same room. Concerning quarantine rooms, 72% of facilities have one. Regarding the acquisition of mice, two-thirds of the institutions acquired the different strains less than five years ago (Figure 1D), but this information is misleading since most facilities bought their breeders from local facilities whose colonies were acquired longer ago. Therefore, if the original provenance of mice is taken into account, these proportions are reversed (Figure 1E), with almost two-thirds of the facilities acquiring their colonies more than 20 years ago. In this sense, 80% of facilities have directly or indirectly acquired their colonies from Jackson or Charles River Laboratories. One-fifth of the institutions produced less than 1,000 mice per year (Figure 1F) and except for one facility that is devoted to quality control, more than 50% of mice produced are employed in research projects. Breeding records are kept in all the facilities except one, consisting of written records in 28% of the cases, and mixed (written and digital) in the remaining institutions. Genetic quality is controlled only in four facilities, three of which are the main mice suppliers to the rest of the institutions mentioned above. Two facilities have sent samples abroad to control the genetic quality, whereas the other two have analyzed their mice colonies in a local laboratory. Although only two establishments mention not following a specific breeding system, the breeding methods for inbred strains/outbred stocks were not properly defined in most of them. It is also interesting to note that two institutions specified that it was difficult for them to maintain the outbred status of their colonies.

### Nutritional status

**Figure 2:**
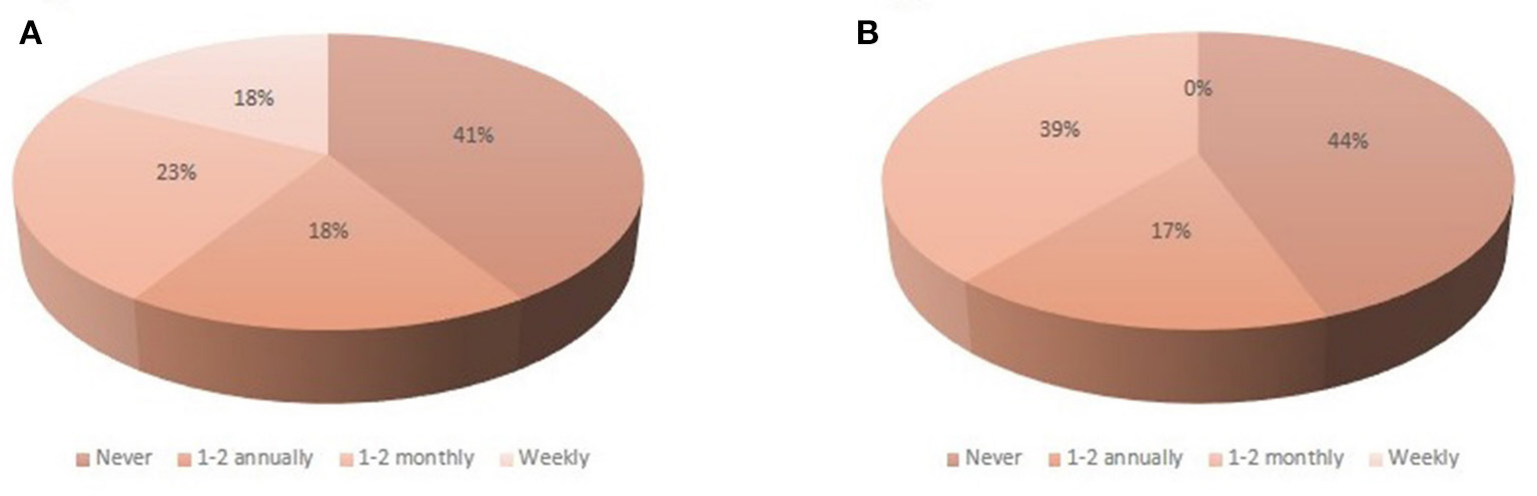
Overview of the situations associated with the supply of water. **(A)** Frequency of flooded cages due to malfunction of water bottles. **(B)** Frequency in which empty bottles are found during cleaning routines.

All the establishments provide *ad libitum* water, with two-thirds of them applying a treatment to reduce the microbiological count. Results concerning the frequency of findings of flooded cages due to malfunction of water systems or of empty bottles are presented in Figure 2A, B, respectively. Regarding feeding, all establishments provide food *ad libitum*, and only half of them apply a treatment to reduce the microbiological count. In 39% of facilities, food supplementation is included as an enrichment strategy to improve breeders' performance, or to compensate for deficiencies in the rodent chow. During the last month, four facilities reported having witnessed body condition below the ideal scoring of 3 and they were able to identify the reason. Also, five facilities declared having found mice with body conditions above this ideal scoring. In Argentina there are two local producers of food for mice and rats: Asociación de Cooperativas Argentinas (ACA) and Grupo Pilar S.A. (GEPSA), and therefore, all the facilities use one of these brands or even a mix of both. At least 10 establishments reported the regular presence of dust in the food, variable characteristics, and even the presence of insects, independently of the brand.

### Environment

**Figure 3:**
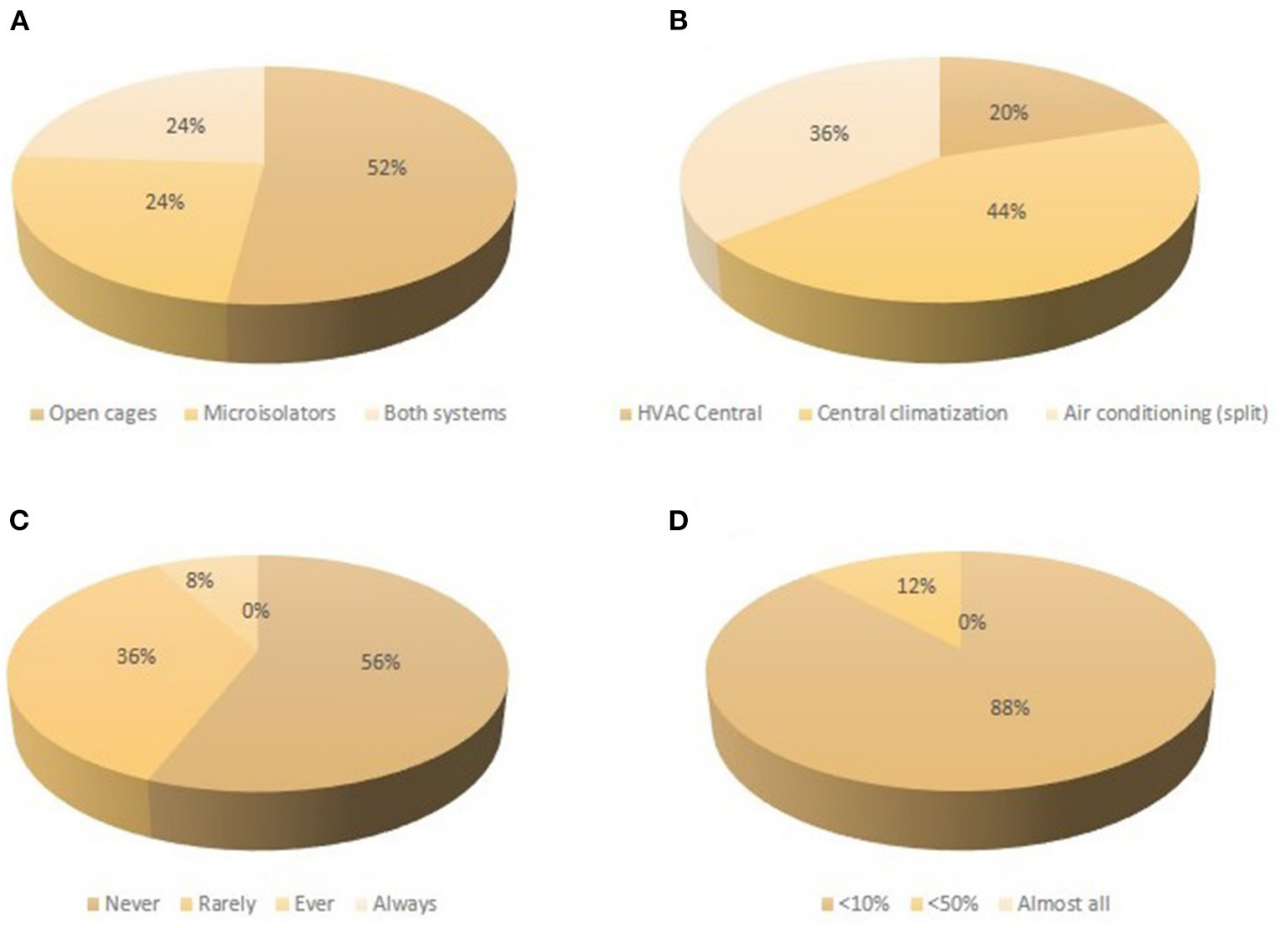
Summary of the environmental conditions that the animals are exposed to. **(A)** Type of cages held in animal facilities. **(B)** Heating, ventilation, and air conditioning (HVAC) systems. **(C)** Frequency of mucous membranes irritation experienced by the personnel when entering the animals' rooms. **(D)** Presence of fully dumped cages when changed.

Half of the institutions maintain mice in opaque cages that impair continuous observation of animals inside the enclosure. According to the different answers, these cages have been and continue to be replaced by transparent ones, but since it implies an important expense, it will still take time to discard the opaque cages. Economic reasons are also at the base of the fact that around 75% of facilities still have open-top cages (Figure 3A). From those establishments with individually ventilated cages (IVC), all except one manipulate animals inside a change station. Lesions in the animals due to the cage design have been noticed in four establishments. Concerning environmental parameters, temperature is maintained constant in all the facilities by means of different systems (Figure 3B), and positive pressure between the rooms and the corridors (a strategy to avoid/reduce microbiological contamination) is maintained in 56% of the establishments. In addition, with the exception of two facilities, all institutions have air extractors installed in the animal rooms, resulting in very few reports of personnel suffering (mucous membranes) irritation caused by ammonia accumulation (Figure 3C). All the establishments work under white light in the rooms. When cleaning the cages, bedding is barely dumped/wet in most cases (Figure 3D). With respect to the bedding, 80% of the establishments use wooden shavings, 16% employ corncob, and the rest, a mixture of both materials. Despite the fact that 76% of the facilities treat the bed material to reduce the microbiological count, only 44% treat the bedding to reduce the dust. Noises in the rooms can be heard from outside in 28% of the facilities. Finally, whereas 68% of the institutions count with an emergency power generator, only 20% of them count with an emergency contingency plan in case evacuation is needed.

### Health status

**Figure 4:**
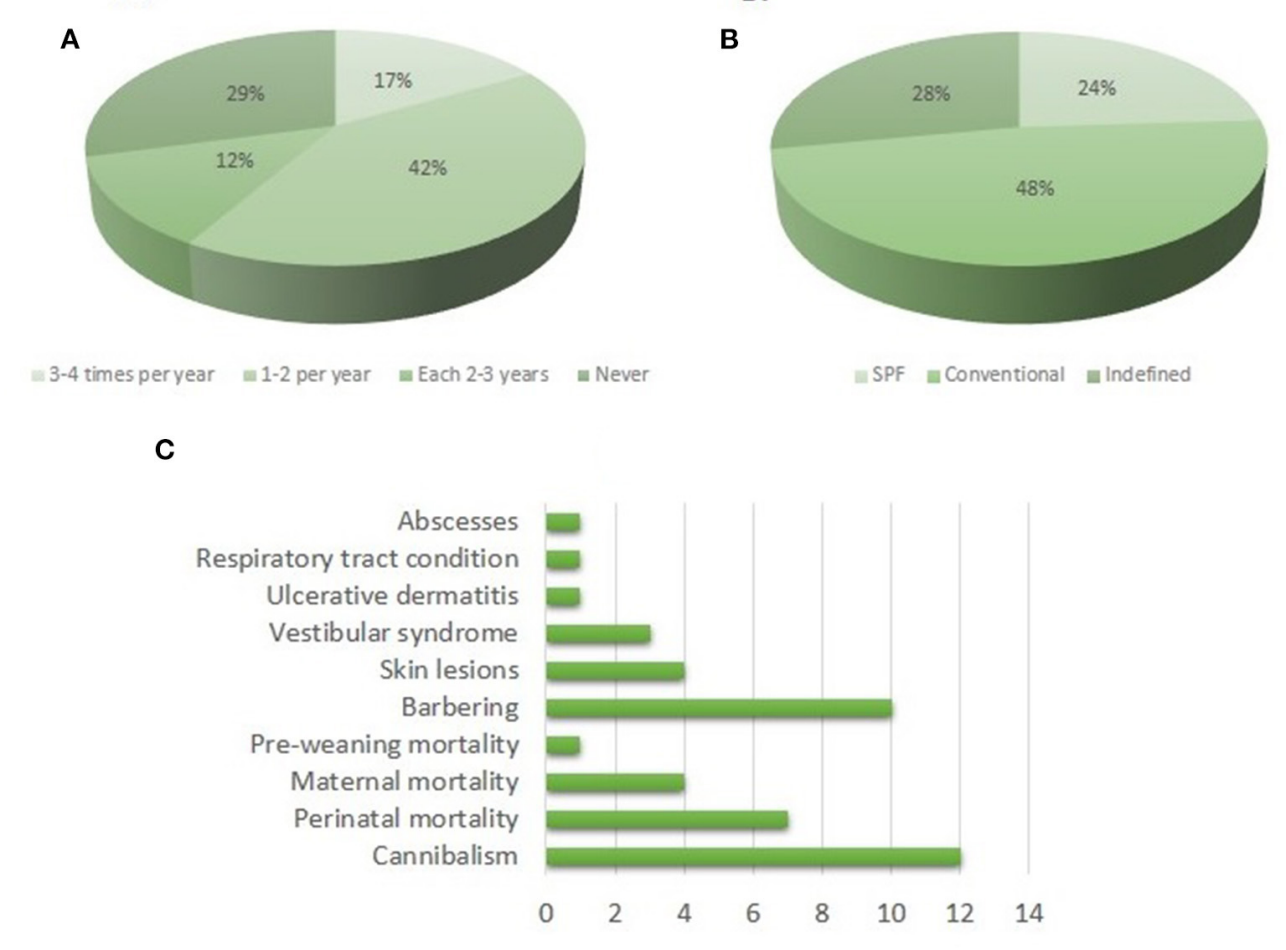
Overview of the health status of the breeding colonies. **(A)** Frequency of microbiological controls. **(B)** Type of microbiological status. **(C)** Prevalence of the common pathological findings.

Although not mandatory, 92% of institutions have an Attending Veterinarian. According to the frequency of monitoring, institutions were grouped as shown in Figure 4A, with almost one-third of facilities not controlling the microbiological status of their colonies. Among the institutions that perform regular microbiological monitoring, six of them send their samples to the Laboratory of Experimental Animals (LAE), Faculty of Veterinary Sciences, National University of La Plata, whereas two of them send their samples abroad (to Charles River Laboratories). All of them assess bacteria, virus, fungi, and parasites. The rest of the establishments (11) analyze their colonies in local laboratories in which not all of the mentioned agents are studied, yielding incomplete microbiological status profiles. Therefore, although six facilities declare themselves as Specific-Pathogen-Free (SPF) and 12 as conventional (Figure 4B), the agents controlled according to their own reports are not adequate to declare that status (13). In 44% of facilities, treatments against parasites are applied, either preventively or after positive results. Concerning adverse situations in mice' cages, the conditions reported more frequently are barbering and cannibalism, followed by perinatal mortality (Figure 4C).

### Behavioral interactions

#### Interactions with the environment

**Figure 5:**
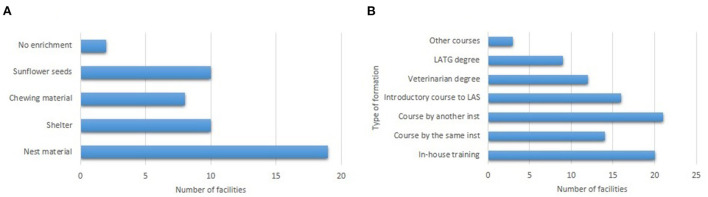
Outline of the factors which mice interact with. **(A)** Type of enrichments that facilities include to the cages of the breeding colonies. **(B)** Type of training that the personnel receives.

Besides bedding, water, and food, enrichment elements are commonly added to the cages as shown in Figure 5A. While 20% of facilities change enrichments depending on the type of animal (usually, increasing enrichment in reproduction cages), 20% of facilities also vary enrichment elements along the year. No institution reported adverse effects due to the incorporation of environmental enrichment. Only three establishments declared having found an animal outside of its cage.

#### Interactions with other animals

Except for three facilities, animal groups were maintained after weaning. Five facilities reported hearing audible vocalizations that may be indicative of fights between animals. Whereas 28% of establishments keep certain mice categories single-housed, during the last month, 32% of them had to separate already established groups due to fighting.

#### Interactions with the people

Sixty percent of the facilities have personnel attending during weekends. With respect to the type of continuous education, distribution is shown in Figure 5B. Ten facilities employ non-aversive methods to manipulate mice, and it is always combined with tail handling. Only in five institutions, the personnel is not stable for the same group of animals. During the last month, no facility has reported any mice biting the personnel.

## Discussion

In the present work, we describe the current state of mouse breeding facilities in Argentina. There are a number of caveats associated with the idiosyncrasy of the country and the restrictions (due to economic and bureaucratic reasons, among other things) that researchers, technicians, and facility managers have to face on a day-to-day basis. Here, we have described the current infrastructure and husbandry of breeding facilities, which are at the core of animal research.

It is important to remark that no international certified breeders (such as Charles River, Jackson, Harlan, or Taconic Laboratories) have facilities neither in our country nor in any other Latin American country. Alternatively, there are three facilities in Argentina that provide mice with certified genetic and microbiologic quality. This fact is key to understanding the dynamics of our facilities, as many of the smaller suppliers have first obtained their breeders from these bigger institutions, a situation already described in a previous report (14). This could also be influencing the low availability of different strains in the country, with most facilities breeding mice from the C57 and BALB/c families. This is in line with the fact that they are the most commonly used strains, but considering that international suppliers are not readily available in the country, it certainly restricts the possibilities of researchers. Indeed, it would be important to envision a plan that brings less profitable strains for specific research protocols that have a national interest (e.g. to study endemic diseases).

From this survey, it was possible to identify that the average animal facility in Argentina uses open-top cages with wood shavings and some sort of nest material. Although this might not be the trend in Laboratory Animal Science (which consists of IVC cages with corncob bedding), there are reports suggesting that these local conditions could be better for animal welfare. For example, the literature suggests that IVC cages can induce chronic cold stress (15), and males housed in these cages (especially when corncob bedding is used) tend to fight more (16). Corncob bedding is usually preferred among animal technologists because it reduces the spread of allergens (17) and the ammonia levels inside the cages (18). Nevertheless, the presentation of high levels of ammonia in the animals' rooms—as perceived by the personnel- is not preponderant among the institutions surveyed, and wood shavings—in comparison to corncob bedding- are always preferred by the mice (19). Moreover, as the presence of dumped bedding in the cages when cleaning is rare, this type of bedding seems to have adequate absorbance. Still, institutions should increase their efforts to improve the quality of the bedding. This can be done relatively easily by sieving the wood shavings (this is currently done in less than half of the facilities) and sterilizing them with any method available.

In accordance with standard practice generally adopted across mouse facilities worldwide, food and water are administered *ad libitum*. Together with the fact that all facilities report the use of standard cages which largely limits the possibility of exercising, the finding of overweight in some facilities could be expected. Paradoxically, underweight mice (with body condition scoring below three) were also detected in several facilities. This is a complicated issue to address since specific diets are not easily available in the country (e.g. low fat), so diet imbalances in some of the animal categories could be expected. This problem is worsened by the fact that some serious quality issues, such as the presence of insects, were noted by some institutions. The presence of empty water bottles is a rare event, but the occurrence of flooding in the cages is relatively common, again potentially due to the quality of the water bottles that are available in the animal facilities.

All institutions use white lights, but given that opaque cages are still widely implemented (half of the institutions) and the fact that some sort of protection from the direct light is usually provided (either by providing nest material or shelter), we do not expect that this condition would be particularly aversive for the mice. Cleaning routines under white light might alter their circadian rhythm (20), but in general, this happens only once a week in breeding facilities. Although using an inverted light cycle can reduce anxiety and improve animal welfare (21), as daily supervision with opaque cages would be even harder under red light, changing the light system should not be one of the priorities for these institutions. On the contrary, it would be better to try to update the cage systems so that they allow unrestricted visualization, avoid physical lesions to the animals, and reduce the possibility of animals escaping.

Concerning the microbiological monitoring of colonies, it is interesting to remark that there is one diagnostic laboratory in Argentina, the Laboratory of Experimental Animals (LAE) (Faculty of Veterinary Sciences, National University of La Plata) that follows FELASA recommendations for the health monitoring of rodents in breeding colonies (13). According to the responses obtained, several facilities screen an incomplete set of microbiological agents. More importantly, 28% of the facilities report that they do not screen against any kind of pathogen, which have implications not only in terms of scientific rigor or animal welfare, but also in the health of the personnel that is potentially at risk of developing zoonosis. The extended use of antiparasitics, both preventively and therapeutically, could be a reflection of this faulty system. Despite this, reported health conditions were relatively infrequent (all but three clinical signs were reported in four or fewer facilities). Two of these three frequent conditions are highly related, as a recent article describes that cannibalism is actually a consequence of perinatal mortality (22). Although we cannot corroborate that this phenomenon is happening with our current data, it might explain the co-occurrence of these undesirable conditions in many facilities. The remaining frequent condition (barbering) has been described as a behavioral problem arising from rearing in standard cages (23).

Supervision of the animals at appropriate intervals is key to guaranteeing their wellbeing (24). Unfortunately, supervision is suboptimal in the surveyed establishments due to the extensive incorporation of opaque cages and the absence of attending personnel during the weekends in 40% of the institutions. One positive finding of this questionnaire is that all institutions mention at least one source of training within their personnel. In this regard, despite the fact that in-house training is very extended (20 out of 25 institutions), all facilities also describe that they outsource their training to other institutions. In addition, some of this training is highly specialized, consisting of veterinarians and people with Laboratory Animal Technologist (LATG) degrees. This is remarkable since there is currently no legislation in the country regulating minimum requirements for the people working with laboratory animals. We believe that this could be ascribed to a long tradition of researchers, technicians, and educators in the field of Laboratory Animal Science (5). Indeed, Argentina is the only country in the region that has a 3-years undergraduate degree for LATG (Técnico Universitario para Bioterios) at the University of Buenos Aires. In this sense, the low rate of incidents with the animals (no biting reported in any of the facilities) is most likely a reflection of the preparation of the people working with them.

Cage fighting is currently one of the primary threats to mice welfare (16), a problem that has also been detected in this survey as several institutions reported that they heard vocalizations compatible with cage fighting. Nevertheless, this number is relatively low (just five reports) and only 32% of the animal facilities describe that they had to separate groups that were maintained stable after weaning in the last month. In the aforementioned article, the authors have identified individually ventilated cages and corncob bedding as the greatest predictors for fighting in the mouse cages (16), two components that are rare among Argentine institutions. Therefore, it would be interesting to study the epidemiology of cage fighting and confirm if the prevalence of agonistic behavior and lesions is compatible with the prevalence reported by American and European institutions (16, 25). However, it should be noted that single-housing is still a relatively common practice in the country, and can be one of the reasons for keeping in-cage aggression low.

All but two institutions provide some kind of environmental enrichment, with nest material being by far the most popular resource. This is unsurprising since it is highly preferred by mice (26) and has widely known benefits such as the reduction of male fighting (27), the improvement of breeding productivity (28), and the reduction of cold stress (29). The absence of adverse effects due to environmental enrichment could be ascribed to the fact that nest materials have virtually no detrimental effects (30). Interestingly, some facilities report varying the type of object throughout the year, which can help to reduce animal boredom (31). Nevertheless, it should be mentioned that this is purely an empirical practice, and we are not aware of previous research that standardizes or validates this procedure.

Uptake of non-aversive handling is still relatively low in the country, with fewer than half of the facilities reporting the use of these methods. Moreover, when employed, it was always combined with tail manipulation. In contrast, a recent survey about non-aversive handling with the majority of the participants from Europe and North America has described that 61% regularly use non-aversive handling (with 35% responding that they use it exclusively and 43% in combination with tail handling) (32). Still, it is important to note that dissemination campaigns to promote the incorporation of non-aversive handling are non-existent in Argentina. The aforementioned survey has highlighted the fact that unfamiliarity with the techniques is one of the causes for not using them (32). Non-aversive handling has many benefits not only in terms of animal welfare and the quality of scientific research (33–35), but also in the performance of the breeding colonies: breeding pairs handled with a tunnel produce, on average, one additional pup at weaning than mice handled by the tail (36). Therefore, a good strategy would be the dissemination of these methods to the scientific community by either the local/regional Laboratory Animals Science Associations or the different Scientific Bodies.

To sum up, the areas in which we see that there is greater space for improvement are recent refinements that can have a direct impact on animal welfare, such as non-aversive handling. These can be improved relatively easily with training programs and modifying the established husbandry. Other structural shortcomings/weaknesses will be harder to address, as they will require the commitment of the politicians to implement specific legislation, the establishment of suppliers that guarantee minimum standards, and the improvement of budgets to invest in animal facilities. All in all, the greatest strength of Argentine animal facilities is in the people that care for the animals on a day-to-day basis, in the large network of ethics committees that oversee animal research, and in the existence of the national record of animal facilities. Although adherence to this registry is voluntary, it is currently the only entity that allows for any kind of networking or action at the institutional level such as the execution of the present work.

## Data availability statement

The raw data supporting the conclusions of this article will be made available by the authors, without undue reservation.

## Author contributions

AR and SD conceptualized the idea and designed the survey. AR compiled the survey online and collected the answers. SD summarized the results and both authors jointly wrote the manuscript. All authors approved the final version of the manuscript.

## Funding

The present work was made possible due to a research grant awarded to SD by Argentina's National Agency of Science and Technology (PICT 2019-03984).

## Conflict of interest

The authors declare that the research was conducted in the absence of any commercial or financial relationships that could be construed as a potential conflict of interest.

## Publisher's note

All claims expressed in this article are solely those of the authors and do not necessarily represent those of their affiliated organizations, or those of the publisher, the editors and the reviewers. Any product that may be evaluated in this article, or claim that may be made by its manufacturer, is not guaranteed or endorsed by the publisher.
