# Supplementary material for: Mouse breeding facilities in Argentina: Current state, challenges, and strengths in relation to animal welfare
Source: Front Vet Sci. 2022 Oct 20;9:1031976. doi: 10.3389/fvets.2022.1031976 (PMC9630945; doi:10.3389/fvets.2022.1031976)
Supplement: Supplementary file 1 [file Data_Sheet_1.PDF]

Supplementary Material

1 Full Translated Survey

General Description

1. Besides mice, which species are you breeding?
2. Which strains of mice does the institution breed?
3. Do you breed transgenic mice?
4. How many breeding rooms does the institution have?
5. How many technicians does the institution have?
6. Are the technicians trained to do one specific labour or do they rotate depending on the day?
7. Do you keep animals from different species in the same room?
8. Do you keep different mouse strains in the same room?
9. Does the institution have a quarantine room?
10. When were the breeders bought?
11. Who was the supplier of these original breeders?
12. How many mice do you produce each year?
13. What proportion of the mice are destined to research projects?
14. Do you keep breeding records? In which format?
15. Did you ever monitor the genetic quality of the colony?
16. If affirmative, please indicate the frequency of these controls
17. If affirmative, please indicate the laboratory to which the samples were sent
18. Do you use specific methods to breed the outbred and/or the inbred colonies?

Nutritional Status

19. Is water administered *ad libitum*?
20. Is water treated to reduce the microbiological count?
21. How frequently do you find at least one flooded cage due to the malfunction of the water bottles?
22. How frequently do you find at least one cage with an empty bottle during the cleaning routine?
23. Is food administered *ad libitum*?
24. Is food treated to reduce the microbiological count?
25. Do you supplement the regular diet?
26. If affirmative, please state the reason.
27. In the last month, have you witnessed any animal below the ideal body condition scoring of 3?
28. If affirmative, could you identify the cause?
29. In the last month, have you witnessed any animal above the ideal body condition scoring of 3?
30. What brand of food do the animals eat?
31. Have you noticed any kind of adverse effect due to the mice' diet?

Environment

32. Do you maintain mice in opaque cages (that do not allow continuous monitoring)?

33. If affirmative, does this apply to all the cages in the colony?
34. Do you keep animals in open-top cages or in individually-ventilated cabinets or cages?
35. If kept in individually-ventilated cabinets or cages, do you change the cages under a clean bench?
36. Have you noticed lesions in the animals due to the cage design?
37. Do you keep the room temperature constant?
38. If affirmative, what kind of system do you use to maintain the room temperature?
39. Do you keep a different pressure between the animals' rooms and the corridors?
40. Do you have air extractors installed in the animals' rooms?
41. When the personnel enters the animals' rooms, do they perceive irritation of mucous membranes due to ammonia excess?
42. Under what kind of lighting conditions do you work with the animals?
43. When cleaning the cages, what is the proportion of the cages that have the bedding completely dumped?
44. What type of bedding do you use?
45. Who is the supplier of the bedding?
46. Is bedding treated to reduce the microbiological count?
47. Is bedding treated to reduce the amount of dust that it may contain?
48. Can you usually hear audible noise from the outside inside the animals' rooms?
49. Does the institution count with an emergency power generator?
50. Does the institution count with an emergency contingency plan in case evacuation is needed?

Health Status

51. Does a veterinarian supervise the facility continuously?
52. Do you send samples to a diagnostic laboratory for microbiological monitoring of the colony?
53. If affirmative, what is the frequency?
54. If affirmative, to which labs do you remit the samples?
55. In those controls, which microbiological agents are assessed?
56. What is the health status of the animal colony?
57. Do you use any kind of preventive treatment against a pathological microorganism?
58. If affirmative, what kind of treatment?
59. Have you witnessed any of the following conditions in the last 6 months?
    - Maternal mortality
    - Perinatal mortality
    - Cannibalism
    - Pre-weaning mortality
    - Skin lesions
    - Barbering
    - Ulcerative dermatitis
    - Respiratory tract condition
    - Diarrhoea
    - Incoordination
    - Vestibular syndrome
    - Abscesses

Behavioural Interactions

Interactions with the environment

60. Besides bedding, water and food, which of the following (if any) are added to the cages?
    Nest material
    Shelter
    Chewing material
    Sunflower seeds
    Other
61. Do you vary environmental enrichment depending on the type of animal? (e.g. breeding females)
62. If affirmative, please describe how do you vary the composition of the environmental enrichment
63. If providing environmental enrichment, do you vary its composition each week?
64. Did you notice any adverse effects due to the incorporation of environmental enrichment?
65. If affirmative, please describe the adverse effects
66. In the last month, have you found any animal outside of the cage?

Interactions with other animals

67. Do you keep groups stable from weaning (or from the time when breeding couples are formed)?
68. Do you frequently hear audible vocalizations that might be indicative of fights between animals?
69. Do you keep single-housed animals in the colony? (e.g. adult males)
70. In the last month, were any of the established groups separated due to fighting?

Interactions with the people

71. Do the personnel attend the institution during the weekends?
72. What type of training did the personnel receive?
    In-house training
    Refresher course by the same institution
    Refresher courses by other institutions
    An introductory course about Laboratory Animal Science
    Laboratory Animals Technologist Degree
    Veterinarian degree
73. How are mice manipulated?
    By the tail using a pair of disinfected tweezers
    By the tail using the hand
    Non-aversively employing a tunnel or a cupped hand
74. Is the person changing cages stable or do personnel rotate among days?
75. In the last month, did you register any incident due to biting?
